# Natural variation of root exudates in *Arabidopsis thaliana*-linking metabolomic and genomic data

**DOI:** 10.1038/srep29033

**Published:** 2016-07-01

**Authors:** Susann Mönchgesang, Nadine Strehmel, Stephan Schmidt, Lore Westphal, Franziska Taruttis, Erik Müller, Siska Herklotz, Steffen Neumann, Dierk Scheel

**Affiliations:** 1 Leibniz Institute of Plant Biochemistry, Department of Stress and Developmental Biology, Weinberg 3, 06120 Halle (Saale), Germany.

## Abstract

Many metabolomics studies focus on aboveground parts of the plant, while metabolism within roots and the chemical composition of the rhizosphere, as influenced by exudation, are not deeply investigated. In this study, we analysed exudate metabolic patterns of *Arabidopsis thaliana* and their variation in genetically diverse accessions. For this project, we used the 19 parental accessions of the Arabidopsis MAGIC collection. Plants were grown in a hydroponic system, their exudates were harvested before bolting and subjected to UPLC/ESI-QTOF-MS analysis. Metabolite profiles were analysed together with the genome sequence information. Our study uncovered distinct metabolite profiles for root exudates of the 19 accessions. Hierarchical clustering revealed similarities in the exudate metabolite profiles, which were partly reflected by the genetic distances. An association of metabolite absence with nonsense mutations was detected for the biosynthetic pathways of an indolic glucosinolate hydrolysis product, a hydroxycinnamic acid amine and a flavonoid triglycoside. Consequently, a direct link between metabolic phenotype and genotype was detected without using segregating populations. Moreover, genomics can help to identify biosynthetic enzymes in metabolomics experiments. Our study elucidates the chemical composition of the rhizosphere and its natural variation in *A. thaliana*, which is important for the attraction and shaping of microbial communities.

In *Arabidopsis thaliana (A. thaliana)*, natural genetic variation has been intensively exploited to study a variety of traits related to plant development, stress response and nutrient content (for review, see Weigel^1^). Several publications have demonstrated that natural variation is a suitable basis for dissecting secondary metabolite pathways by using genetic mapping analyses. The genetics of glucosinolates and its link to pathogen and herbivore resistance have been investigated thoroughly^2^,^3^,^4^,^5^. A large variation of glucosinolates in leaves and seeds was observed for 39 genetically diverse Arabidopsis accessions^6^. Houshyani *et al*.^7^ found that natural variation of the general metabolic response to different environmental conditions is not necessarily associated with the genetic similarity between nine accessions.

Many metabolomics studies focus on aboveground plant tissues. As a result, only limited information is available with regard to the metabolism of belowground parts of the plant.

Roots are crucial for the uptake of water and nutrients. For example, Agrawal *et al*.^8^ utilized natural variation of *A. thaliana* to identify malic acid as a key mediator for nickel tolerance. To communicate with the belowground environment, plant roots also exude metabolites such as flavonoids, phenylpropanoids and glucosinolates^9^, which can attract microorganisms or increase the resistance against pathogens^9^,^10^,^11^. These interactions take place in the rhizosphere, which is regarded as the space adjacent to roots^12^. As the properties of the rhizosphere differ strongly from the bulk soil in terms of microorganism abundance^13^, as well as the qualitative and quantitative metabolic composition^14^,^15^, investigations on root exudates are needed to assess the role of this microenvironment. Micallef *et al*.^16^ demonstrated that the rhizobacterial community composition is influenced by varying exudation profiles.

Non-targeted metabolite profiling of secondary metabolites by liquid chromatography coupled to mass spectrometry (LC/MS) is an ideal analytical platform to link natural metabolite variation to biosynthetic pathways. It allows for the detection and quantification of semipolar compounds^17^, when the resulting three-dimensional signals with a specific mass-to-charge (*m/z*) ratio, retention time (RT) and intensity, so-called features, can be annotated. Depending on the nature of the compound, they are more likely to be detected upon electrospray ionization in the positive (ESI(+)) or negative mode (ESI(−)).

Our approach to investigate natural genetic variation of secondary metabolism in root exudates focuses on 19 *A. thaliana* accessions, which show a large degree of geographic and phenotypic diversity (Supplementary Table S1) and were used to generate the Multiparent Advanced Generation Inter-Cross (MAGIC) lines^18^. Whole genome sequencing revealed that the parental accessions and the MAGIC lines represent most of genetic variability of *A. thaliana* and therefore provide a valuable resource for genetic and metabolic studies^19^,^20^.

The aim of this study is to find out if the root exudate composition in *A. thaliana* is genetically determined. For this purpose, we analysed which metabolites show natural variation, if similar metabolic phenotypes share a genetic base, in particular, if certain characteristics can be traced back to single nucleotide polymorphisms and hence, directly link phenotype and genotype.

## Results

### Non-targeted metabolite profiling of root exudates reveals distinct metabolic phenotypes for 19 Arabidopsis accessions

**Figure 1:**
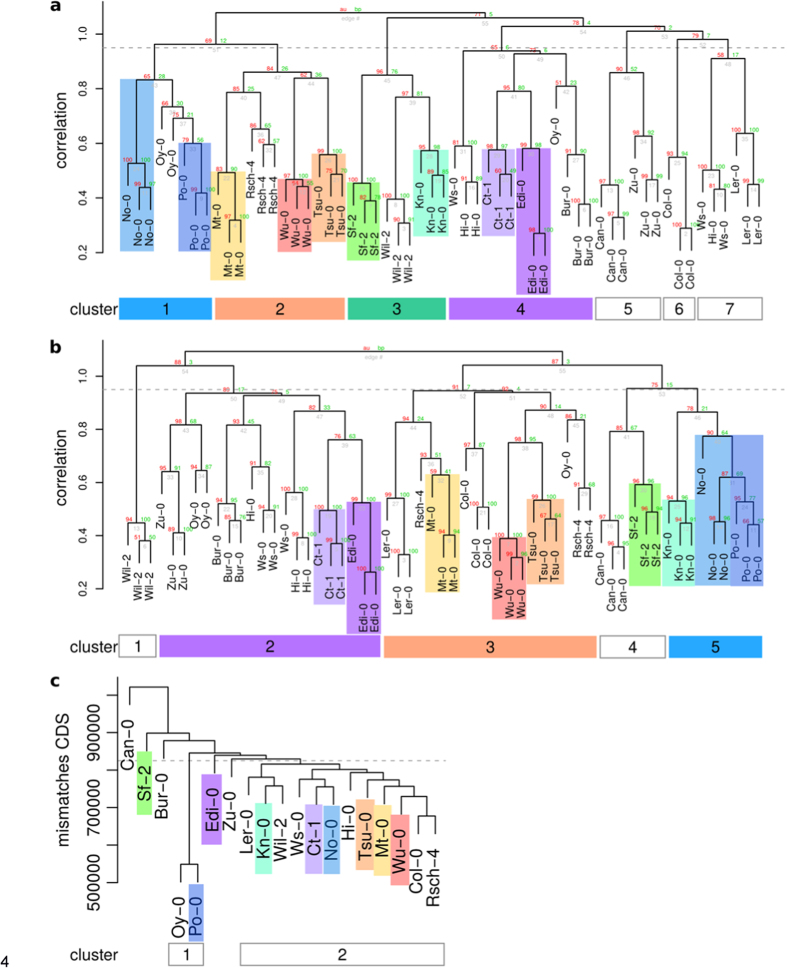
Hierarchical clustering of metabolic features from (**a**) exudates ESI(−), (**b**) ESI(+) and of (**c**) genetic distances. (**a+b**) Features were obtained by UPLC/ESI(−)-QTOF-MS (a) or UPLC/ESI(+)-QTOF-MS (**b**) from exudate samples and differed from the blank (Welch test, p < 0.05). Intensities were corrected for batch effects using SVA and subjected to average linkage clustering with correlation as a distance measure. (**c**) Variant tables of the 19 genomes project were reduced to coding regions, as annotated by TAIR. The sum of all mismatches was used as a distance matrix for average linkage clustering. Dendrograms were cut at a correlation threshold of 0.95 (dashed line). As cluster numbers were not comparable, consistent clusters were coloured across ion modes as a visual guidance.

A clustering analysis was performed to find similarities between the metabolic profiles and sequence polymorphisms of the 19 founder accessions of the MAGIC population of *A. thaliana*. The dendrograms calculated from the metabolic features show a clear separation of accessions in Fig. 1a for exudates measured in ESI(−) and Fig. 1b in ESI(+). At a correlation threshold of 0.95 (dashed line), seven and five clusters, respectively, were observed.

No-0 and Po-0 (blue) were found in the same cluster (cluster 1, ESI(−); cluster 5 ESI(+)) in both ion modes. Ct-1 and Edi-0 (purple) also displayed high similarity in their metabolic profiles. Sf-2 and Kn-0 (green) were in close proximity and would have been in the same clade when cutting the ESI(+) dendrogram at a different threshold. Similar metabolic phenotypes were also detected in the exudation patterns of Wu-0 and Tsu-0, and additionally Mt-0 (orange). These three accessions either clustered in dendrogram branch 2 (ESI(−)) or 3 (ESI(+)).

In both metabolic dendrograms, one Oy-0 sample was observed as an outlier, which did not cluster with the other replicates of Oy-0. For Hi-0 and Ws-0, mixed clusters were observed. The positive ion mode generally harboured more outliers. As obvious from the quality control plots in Supplementary Fig. S1, the outlying samples did not show any extreme deviations on the technical side and were therefore not excluded from further analysis^21^.

For the analysis of genetic diversity, sequence polymorphisms in coding sequences (CDS) extracted from the 19 genomes project^22^ were used for a genetic clustering (Fig. 1c). One large dendrogram branch (L*er*-0, Kn-0, Wil-2; Ws-0, Ct-1, No-0; Hi-0, Tsu-0, Mt-0, Wu-0, Col-0, Rsch-4) had less than 825,000 mismatches (dashed line) while the outliers Bur-0, Sf-2, and Can-0 had increasing numbers of polymorphisms. Oy-0 and Po-0 formed a small cluster and were found in proximity to Edi-0, Zu-0 and the large dendrogram branch.

The metabolic analysis was based on a non-targeted metabolite profiling approach considering metabolic features characterised only by their *m/z* ratios, RTs and intensities. These characteristics are not sufficient to investigate the underlying molecules, its biosynthetic pathway and its potential in plant signaling. Annotations and identifications of metabolites, as shown in the next paragraph, are required to interpret non-targeted metabolic profiles in the biological context.

### Semipolar secondary metabolites are the major components of the exudation patterns

**Figure 2:**
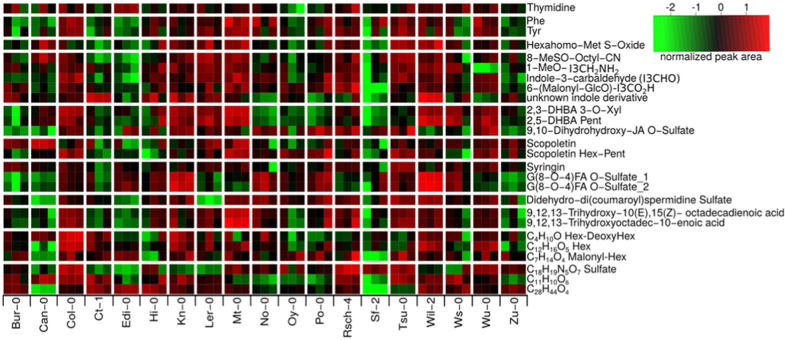
Colour-coded intensity matrix of differential metabolites occurring in exudates. Integrated peak areas were log-transformed and scaled to zero mean and standard variance. A Welch-test was used to find differentially abundant metabolites between the 19 accessions.

Only 25 and 22 of the metabolic signals (455 (ESI(−)), 475 (ESI(+), respectively) could be assigned to metabolites which have been previously described as exudate-characteristic for Col-0^15^. Differential metabolites were detected by a generalized Welch-test between the 19 accessions; their colour-coded intensity map is shown in Fig. 2. Chemically related compounds were placed in groups separated by horizontal spacing.

Among the differential metabolites, there were several compounds with an aromatic moiety, such as the nucleoside thymidine and the amino acids Phe and Tyr. The amino acid derivative hexahomo-Met *S*-oxide had low abundance in the exudates of Sf-2 and was enriched in Mt-0.

A range of glucosinolate degradation products was characteristic for the exudates of some accessions. Edi-0 had rather low levels of indolic compounds and the isothiocyanate hydrolysis product of 8-MeSO-Octyl glucosinolate. Wu-0 showed a clear absence of the neoglucobrassicin (1-MeO-I3M) hydrolysis product 1-methoxy-indole-3-ylmethylamine (1-MeO-I3CH_2_NH_2_), while Sf-2 was missing the malonyl-glucoside of 6-hydroxyindole-3-carboxylic acid (6-(Malonyl-GlcO)-I3CH_2_CO_2_H). An unknown indole derivative (C_10_H_9_NO_3_) was highly abundant in the exudates of Ct-1 and Wil-2, and lowly abundant in Sf-2. Generally, large amounts of the glucosinolate precursor and hydrolysis products were detected in the exudates of L*er*-0, Mt-0 and Wil-2.

Plant hormone-derived metabolites also differed between the 19 accessions. Two salicylic acid (SA) catabolites, 2,3 and 2,5-dihydroxybenzoic acid (DHBA) pentosides, were highly abundant in Col-0, Kn-0, L*er*-0, Mt-0, Wil-2, Ws-0 and Wu-0. No preference for the 3′ or 5′ hydroxylated variant of DHBA was noticed, and both isomers correlated positively with a Pearson correlation of 0.91. 9,10-dihydrohydroxy jasmonic acid (JA) *O*-sulfate was another differential plant hormone catabolite in *A. thaliana* exudates with low levels in Bur-0, Can-0 and Zu-0 and high levels in Col-0, Kn-0, Po-0, Rsch-4 and Wu-0.

Among the phenylpropanoids, the coumarin scopoletin and its glycosides differed in the exudates of the 19 accessions. A hexose-pentose conjugate of scopoletin as well as three other glycosides (C_4_H_10_O Hex-DeoxyHex, C_12_H_16_O_5_ Hex, C_7_H_14_O_4_ Malonyl-Hex) were among the differentially abundant metabolites which were described for Col-0 exudates^15^.

Other differential phenylpropanoids include the monolignol glucoside syringin as well as both isomers of the sulfated dilignol G(8-*O*-4)FA *O*-sulfate consisting of coniferyl alcohol (G) and ferulic acid (FA): it was present at high levels in Kn-0 and Wil-2 exudates. Two hydroxylated fatty acids also showed natural variation and were highly abundant in Mt-0.

Several isoforms of known glycosylated metabolites (e.g. kaempferol triglycosides with *m/z* 739.21) were detected at different RTs indicating differences in sugar conjugation. The investigation of these putatively annotated metabolites can be facilitated by exploring polymorphisms in genes encoding their biosynthetic enzymes.

### The absence of an indolic glucosinolate hydrolysis product and a hydroxycinnamic acid conjugate is genetically determined

Wiesner *et al*.^23^ reported that the accession Wu-0 lacks the 1′-methoxylated indolic glucosinolate due to a premature stop codon in the *CYP81F4* gene^24^. Its frameshift mutation leads to a loss of function and subsequently to the absence of 1-MeO-I3M in roots and leaves^23^, and also its amine, 1-MeO-I3CH_2_NH_2_, in the exudates of our hydroponic system.

**Figure 3:**
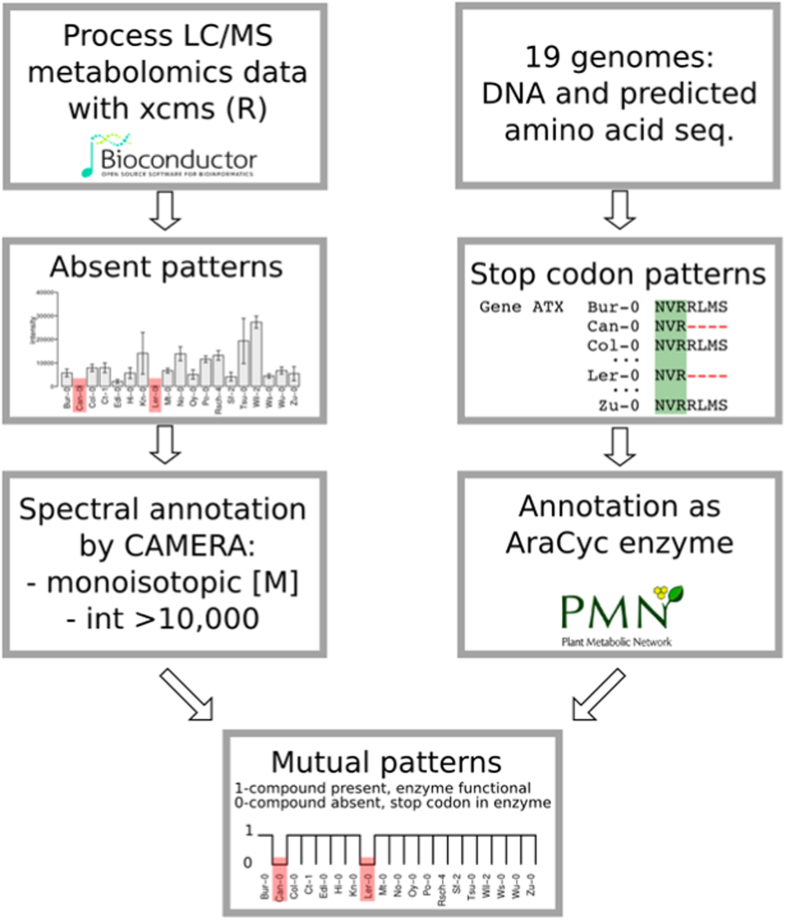
Workflow for matching metabolic patterns of absence with stop codons in genes annotated as AraCyc enzymes. For the metabolic data, 384 out of 455 metabolic features from the ESI(−) data set were absent in at least one accession. 38 of them were annotated as monoisotopic peak [M] by CAMERA. Approximately 32,000 stop codons were detected. 1,588 of AraCyc enzyme-encoding genes displayed a prematurely ended amino acid sequence possibly representing non-functional enzymes that can be causative for metabolite absence.

To elucidate if further metabolite absences in the exudates like 1-MeO-I3CH_2_NH_2_ in Wu-0 can be traced back to a single gene, we developed a workflow to link genomic and metabolic patterns (Fig. 3). Features with the same absence pattern could be different molecular species of the same compound (adducts, isotopes, fragment or cluster ions). Alternatively, they may be different isomers from the same biosynthetic pathway with a common precursor.

**Figure 4:**
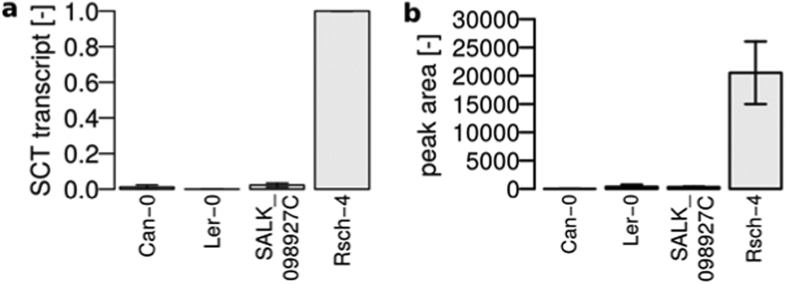
Natural and T-DNA insertion knockouts of SCT. (**a**) Relative transcript levels of SCT in root tissue as determined by qPCR, PP2A as reference, normalized to Rsch-4, mean ± s.e.m., n = 3. (**b**) Peak area counts of cyclic didehydro-di(coumaroyl)spermidine sulfate in exudates, mean ± s.e.m., n = 3.

Among the seven metabolic features with absence in two accessions, three were characteristic for Can-0 and L*er*-0. The hydroxycinnamic acid polyamine derivative cyclic didehydro-di(coumaroyl)spermidine sulfate previously identified in Col-0^15^ and also detected in other accessions was clearly absent in Can-0 and L*er*-0 (Fig. 2). This compound with RT = 3.6 min was absent in the negative ion mode as [M-H]^−^ adduct with *m/z* = 514.17 and [M-2H + Na + CH_2_O_2_]^−^ adduct with *m/z* = 582.15. Another compound with *m/z* = 514.17 eluting at 4.2 min was also absent in Can-0 and L*er*-0. Tandem mass spectrometry (MS/MS) analysis revealed a sulfur trioxide loss in the fragmentation pattern similar to the sulfated cyclic didehydro-di(coumaroyl)spermidine conjugate. Can-0 carries a premature stop codon in the gene AT2G25150 encoding spermidine dicoumaroyl transferase (SCT), whereas in L*er*-0, a large deletion is present in the CDS of this gene^22^. Both accessions have no detectable levels of SCT transcript in their roots (Fig. 4a).

Thus, neither Can-0 nor L*er*-0 possess SCT activity to most likely produce cyclic didehydro-di(coumaroyl)spermidine sulfate and its isomer. To further support the data observed with these two accessions, we analysed the exudates of the homozygous knockout line SALK_098927C (Col-0 background), which indeed did not display any peaks with *m/z* 514.17 ESI(−) at 3.6 min, as shown in Fig. 4b, and thus confirm our hypothesis.

The above results for the Wu-0 and Can-0/L*er*-0 pattern showed the feasibility of such an association analysis to link compounds to their biosynthetic pathways. In specific cases, there is a direct connection between metabolic phenotype and genotype. Therein, metabolite variation among Arabidopsis accessions can be traced back to individual SNPs without trait segregation and QTL mapping.

### Matching metabolic and genetic patterns can indicate compound class

Genetic alterations may be exploited to characterise so far unknown compounds which are part of related biosynthetic pathways^25^. MS/MS fragmentation facilitates the annotation of chemical substructures, which are often characteristic for a certain class of compounds. Knowledge about biosynthetic pathways can further support the assignment of unknown features to compound classes.

For the annotation of metabolites, collision-induced dissociation (CID-) MS was performed for 17 selected MS1 ESI(−) features obtained by the above described screening.

With the help of MS/MS spectra, nine out of 17 features were annotated and for five further features, the elemental composition was determined. An overview of compounds, fragment spectra and matching enzymes is given in Supplementary Table S5.

**Figure 5:**
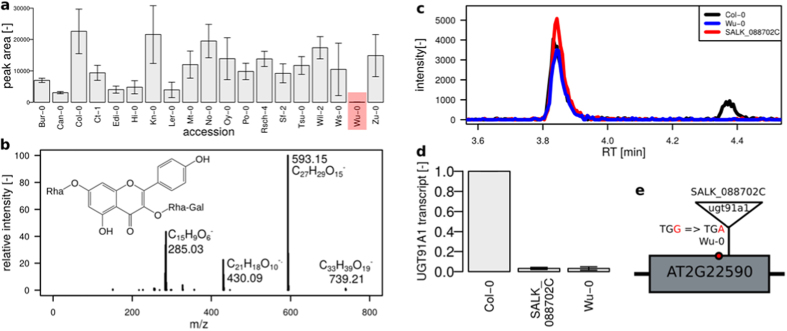
Robinin absence is linked to a stop codon in the UGT91A1 encoding gene. (**a**) Peak area counts, mean ± s.e.m. (n = 3) with absence in Wu-0 (highlighted in red) (**b**) MS/MS spectrum of robinin, 30 eV, (**c**) extracted ion chromatogram at *m/z* 739.21 with kaempferol 3-*O*-Rha(1→2)Glc 7-*O*-Rha eluting at 3.9 min and the galactose-conjugated robinin eluting at 4.3 min not detected in the natural knockout Wu-0 and T-DNA insertion line SALK_088702C, (**d**) relative transcript levels of UGT91A1 in roots as determined by qPCR, PP2A as reference, normalized to Col-0, mean ± s.e.m., n = 4, (**e**) schematic representation of the UGT91A1 gene (one exon) and the loss-of-function mutations in Wu-0 and SALK_088702C.

A compound (*m/z* 739.21, RT = 4.3 min) that was not found in the exudates of Wu-0 (Fig. 5a) was identified as a flavonoid with the same elemental composition (C_33_H_40_H_19_) and fragment spectrum as kaempferol 3-*O*-Rha(1→2)Glc 7-*O*-Rha^15^. The RT shift indicates different glycosidic conjugation. This compound was identified as robinin (kaempferol 3-*O*-Rha-Gal 7-*O*-Rha) by an authentic standard having a galactose moiety instead of glucose in the diglycoside at the 3′ position (Fig. 5b). One out of the 16 premature stop codons characteristic for Wu-0 was present in AT2G22590.1, which encodes the UDP-glycosyltransferase (UGT) superfamily protein UGT91A1. This gene is coexpressed with the flavonol synthase 1 (FLS1, AT5G08640) and chalcone flavanone isomerase (TT5, AT3G55120) encoding genes that are annotated with the “flavonoid biosynthetic process” by Gene Ontology^26^. The exudates of the homozygous knockout line SALK_088702C (Col-0 background) were missing robinin and its UGT91A1 transcript levels in roots were diminished (Fig. 5c–e).

The hydroxylated fatty acid 9,12,13-trihydroxyoctadec-10-enoic acid (9,12,13-TriHOME, KEGG C14833) was not present in the exudates of Edi-0 and Zu-0 (Fig. 2). Its lack corresponds to a SNP pattern introducing a stop codon into a long-chain-alcohol *O*-fatty-acyltransferase gene (AT5G55360.1). The unsaturated variant 9,12,13-trihydroxyoctadec-10(E),15(Z)-enoic acid, however, could be detected in Edi-0 and Zu-0 exudates, but not in the Ct-1 accession, and accordingly, pointed to different polymorphism patterns. Besides, similar intensity distributions of both hydroxylated fatty acids were found across the exudates of the 19 accessions (Fig. 2).

These examples show that the direct search for a metabolite-enzyme-connection can provide valuable insights into biosynthetic pathways but require careful examination of the resulting candidate genes.

## Discussion

This study showed how the exudation pattern of *A. thaliana* accessions is reflected by a genetic clustering of polymorphisms in their CDS. The previously reported similarity of the German and Norwegian accession Po-0 and Oy-0^22^ was only observable at metabolic level in the ESI(−) dendrogram. The close relation was confirmed by the genetic clustering. However, we also observed closely related metabolic profiles of Po-0 with No-0 (blue), which has not been described before. Neither the metabolic proximity of Sf-2 and Kn-0 (green) nor of Ct-1 and Edi-0 (purple) were reflected by small genetic distances.

The similarity of the Wu-0, Tsu-0 and Mt-0 was present in both ESI dendrograms of the exudate analysis and seems to be genetically determined. The close genetic relation between the Japanese accession Tsu-0 and Mt-0 from Libya has already been reported by Nordborg *et al*.^19^ as well as by De Pessemier *et al*.^27^, and was confirmed for metabolic exudate and the CDS profiles (orange).

The clustering of metabolic profiles demonstrated that genetic variation between the 19 founder accessions of the Arabidopsis MAGIC population is indeed reflected in the exudate metabolome. This is in contrast to the previously reported only minor correlation between shoot metabolic and genetic similarity^7^ of nine accessions, partially overlapping with the MAGIC founder lines. Compared to 149 SNPs that were used to estimate a genetic distance by Houshyani *et al*.^7^, our analysis included 640,066 polymorphisms that were exclusively within CDS. The usage of SNPs in CDS ensures a comprehensive, but most direct genotype-phenotype-association, disregarding regulatory sequences. From hierarchical clustering, we can conclude that the three dendrograms reflect the genetic determination of the exudation profile of several Arabidopsis accessions. Both, the genetic and thus the metabolic profiles, may have been affected by selection processes at the collection sites^25^. Information on environmental conditions, especially characteristic rhizosphere data of the original locations, would be of great interest, but unfortunately, these are not well documented^28^.

In our study, a variety of glycosylated and sulfated compounds are the key metabolites that underlie natural variation in the exudates of the MAGIC parental lines. Scopoletin was found both as an aglycone and hexose-pentose conjugate. However, glucosinolates were only detected as degradation products (amines, carbaldehydes, isothiocyanates). Currently, we cannot elucidate whether glucosinolate exudation is initiated by myrosinase activation or if hydrolysis was caused by the sample preparation procedure.

Previously, hormones were described as constituents of root exudates^29^. Despite that, plant hormones were difficult to detect with the analytical method due to their low abundance. Plant hormone-derived metabolites were detected as glycosylated and sulfated in case of SA and JA, respectively. Natural variation is reflected by a great spectrum of glycosidic conjugation. This was shown for SA catabolites. SA was present in the exudates of Col-0 in the study of Strehmel *et al*.^15^ but did not pass their stringent filtering criteria to be included in their exudate compound collection, while SA derivatives with 2,3 or 2,5- dihydroxy-substituted benzoic acid pentose conjugates passed the filter. As shown in Supplementary Fig. S2, high amounts of SA were found in Kn-0, Wil-2 and Wu-0, the lowest amount was present in Sf-2 exudates, one of the accessions with also low DHBA pentoside levels. Interestingly, solely pentosides but no hexosides of DHBA were detected in the root exudates of Col-0^15^. Li *et al*.^30^ investigated the discrimination of hexose and pentose conjugation in 96 *A. thaliana* accessions. Combined QTL and association mapping pointed to a locus on chromosome 5 within proximity of a gene encoding a putative UGT with pentose specificity. The findings of this study support the previously reported low ratio of pentose-hexose conjugates for Edi-0^30^. Sf-2 was the accession with the lowest DHBA pentoside-hexoside ratio, which may be caused by a non-functional pentose-conjugating UGT and a background hexose-transferase activity that leads to a DHBA hexoside phenotype.

**Figure 6:**
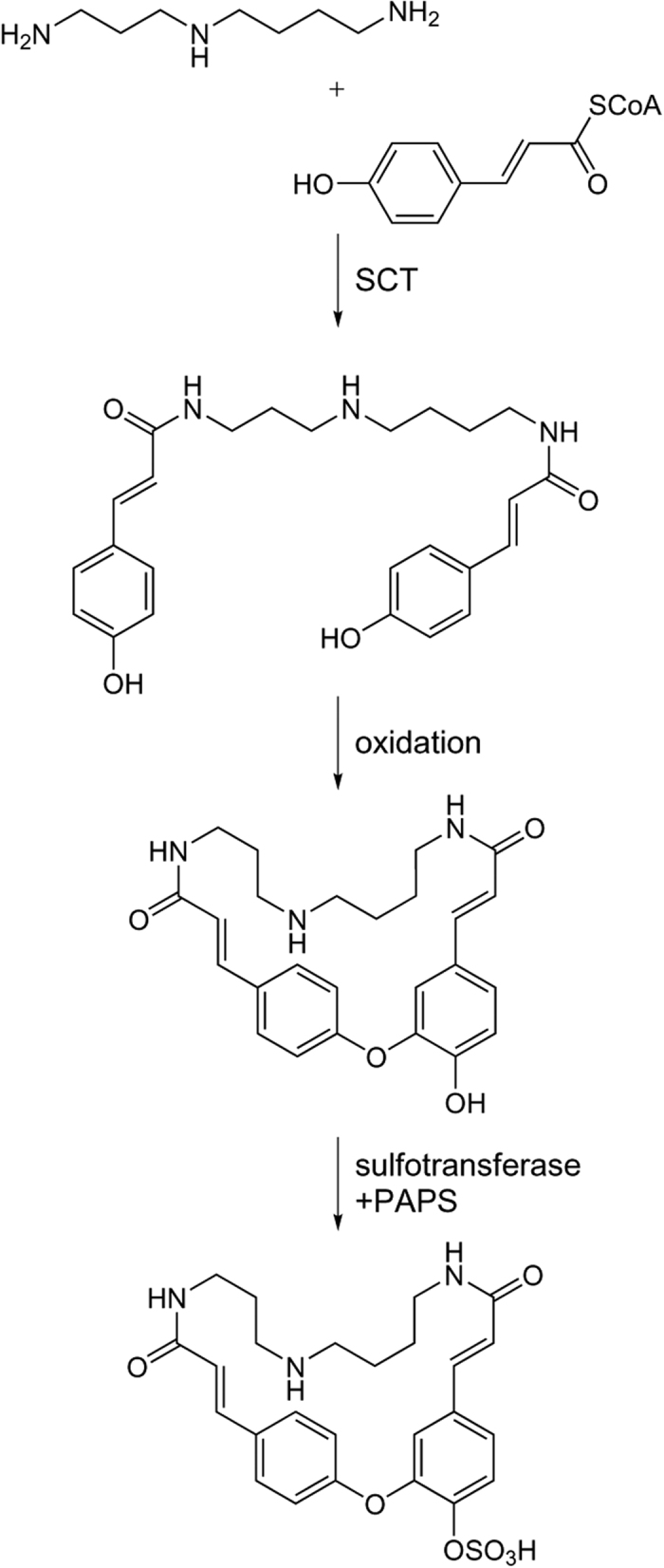
Biosynthetic pathway of cyclic didehydro-di(coumaroyl) spermidine sulfate. Di(coumaroyl)spermidine is synthesized by SCT^47^ and subsequent oxidative ring closure and sulfonylation leads to cyclic didehydro-di(coumaroyl) spermidine sulfate, PAPS = 3′-phosphoadenosine-5′-phosphosulfate.

Chemically related compounds often derive from the same biosynthetic pathway. The characterisation of these metabolites might be facilitated by combining metabolic patterns with genomic data. Thus, an analysis workflow was developed which compares metabolite and sequence polymorphism patterns. In order to reduce the complexity, qualitative metabolic patterns were extracted and compared with the presence of premature stop codons in enzyme-encoding genes. The absence of a sulfated cyclic di(dehydrocoumaroyl)-spermidine was traced back to a single genomic alteration diminishing SCT activity in Can-0 and L*er*-0. These data support the hypothesis postulated by Strehmel *et al*.^15^ that the cyclic conjugate is derived from di(coumaroyl)spermidine synthesized from spermidine and coumaroyl-CoA by SCT as illustrated in Fig. 6. A subsequent oxidative ring formation and sulfonylation led to sulfated cyclic di(dehydrocoumaryol)-spermidine^31^. Nevertheless, the coumaroyl spermidine transferase activity can hardly be inferred from the gene annotation as “HXXD-type acyl transferase family protein”. This workflow furthermore pointed towards the substrate specificity of UGT91A1. Previous studies have shown that UGT91A1 is regulated by MYB transcription factors and speculated about its involvement in glycosylation of flavonols or flavonol glycosides^32^. We could show that in the absence of UGT91A1 enzymatic activity no galactose transfer to kaempferol 3-*O*-Rha 7-*O*-Rha (kaempferitrin) is catalysed to produce robinin. However, the presence of the glucose-substituted isomer kaempferol 3-*O*-Rha(1→2)Glc 7-*O*-Rha implies the involvement of a different UGT not accepting galactose but rather glucose as a substrate. We hereby found that UGT91A1 might have similar flavonoid substrate specificity as UGT73C6 and UGT78D1^33^. However, the patterns of two closely related hydroxylated fatty acids did not show mutual absences. Their intensity distributions were similar and point out the threshold issue in the absence definition. The SNP in AT5G55360 is likely to be a false positive candidate that needs to be excluded by a careful interpretation.

Future investigations will focus on the refinement of our approach by addressing the following points: i) When is a peak defined as absent? We relied on the decision of the peak-picking method centWave^34^ in the xcms package^35^. If the algorithm found a peak at a particular *m/z* and RT in one accession but could erroneously not match its peak criterion in any replicates of another accession, the peak was defined as absent. ii) For a proof of concept, our workflow only included nonsense mutations in CDS of single genes. More complex studies would include amino acid exchanges in CDS, alterations in promoter regions as well as cases of gene function redundancies.

Linking stop codons with metabolite absences helps with the elucidation of secondary metabolite pathways but still requires fragment spectra to be interpreted manually and gene annotations have to be carefully checked for a possible involvement within the biosynthetic pathway of the metabolite. The connection has to be validated by knockout lines of the respective candidate genes.

Our study revealed natural variation in the root exudate composition of 19 genetically diverse accessions of *A. thaliana*. Combining nonsense mutations with metabolic patterns of the exudates facilitated to determine the genetic base of specific metabolite absences. Furthermore, the integration of sequence data can help to identify compound classes in metabolomics experiments. Our study can aid to further unravel biochemical and molecular processes in the rhizosphere by providing a metabolomics resource of root exudates (MetaboLights, accession number MTBLS160, http://www.ebi.ac.uk/metabolights/MTBLS160). Future investigations should aim at correlating metagenomics with exudation profiles in order to deduce characteristics that can be exploited to circumvent limiting abiotic factors and decrease the susceptibility towards biotic stresses.

## Methods

### Plant material

Seeds of the accessions Bur-0, Col-0, Can-0, Ct-1, Edi-0, Hi-0, Kn-0, L*er*-0, Mt-0, No-0, Oy-0, Po-0, Rsch-4, Sf-2, Tsu-0, Wil-2, Ws-0, Wu-0, and Zu-0 of *A. thaliana* (Supplementary Table S1) were obtained from the European Arabidopsis Stock Centre. The T-DNA insertion lines SALK_098927C and SALK_088702C were obtained from the SALK institute and Dr. Ralf Stracke (Bielefeld), respectively, and characterised as elaborated in the Supplementary Methods.

### Plant cultivation

All seeds were surface-sterilized prior to plant cultivation. Then, all lines were cultivated in a hydroponic system with three independent biological experiments as previously described^15^ and in the Supplement. Culture medium was used as a blank. Medium was collected after one-week-exudation (week 5–6) and resulted in 57 pooled root exudates (of four plants each).

### Sample preparation

Root exudates were prepared according to Strehmel *et al*.^15^ and as described in Supplementary Methods.

### Non-targeted metabolite profiling analysis

Changes in metabolism were analysed by ultra-performance liquid chromatography coupled to electrospray ionization quadrupole time–of–flight mass spectrometry (UPLC/ESI-QTOF-MS) according to Böttcher *et al*.^36^.

All mass spectra were acquired in centroid mode and recalibrated on the basis of lithium formate cluster ions.

A detailed description of plant cultivation, sample preparation and metabolite profiling can be found in Supplementary Methods.

### Data analysis

Raw data files were converted to mzData using CompassXPort version 1.3.10 (Bruker Daltonics 4.0). Subsequently, the R package xcms version 1.41.0^35^ was used for feature detection, alignment and filling of missing values. On this account, features were detected with the help of the centWave algorithm according to Tautenhahn *et al*.^34^ (snthr = 5, scanrange = c(1,3060), ppm = 20, peak width = c(5,12)), matched across samples (xcms function group, minfrac = 0.75, bw = 2, mzwid = 0.05, max = 50), corrected for retention time shifts (method = “loess”) and grouped again. Missing values were imputed with the xcms function fillPeaks which integrates raw chromatographic data. The data matrix was extracted using the diffreport function.

DataAnalysis 4.0 (Bruker Daltonics) was used for generation of extracted ion chromatograms, deconvolution of compound mass spectra and calculation of elemental compositions. For relative quantification of compounds extracted ion chromatograms from the non-targeted analysis were integrated with QuantAnalysis 2.0 (Bruker Daltonics) using the quantifier ions as listed in Supplementary Table S3. Peak areas were log-transformed and z-scaled to achieve normal distribution. Differential metabolites were detected by a generalized Welch-test between the 19 accessions (unequal variances, one-way layout, p < 0.05, corrected for multiple testing by Benjamini-Hochberg’s method^37^).

All statistical procedures were performed with the R statistical language version 3.0.0^38^ and the Bioconductor environment^39^. All data are available from the MetaboLights repository under the accession number MTBLS160 (see Supplementary Methods).

### Hierarchical clustering

Before hierarchical clustering, remaining missing values were replaced with half of the minimum feature intensity. Feature intensities were logarithmized, z-transformed and checked for normality with a Kolmogorov-Smirnow test. Non-biological sources of variation were removed by surrogate variable analysis from the SVA package version 3.8.0^40^. In order to discriminate between experimental artifacts and metabolic features in the non-targeted analysis, a generalized Welch test (unequal variances, one-way layout) was applied to find differential features (p < 0.05, corrected for multiple testing by Benjamini-Hochberg’s method^37^) between the 19 accessions and blank. As a post-hoc test, 2-sample Welch tests were used to find features that were differential (p < 0.05) from the blank in at least one accession. This resulted in 455 out of 1950 ESI(−) and 475 out of 3738 ESI(+) metabolic features used for hierarchical clustering. Hierarchical clustering was performed via multiscale bootstrap resampling with the R package pvclust version 1.2–2^41^, which improves robustness by providing an approximately unbiased p-value (AU, red number in Fig. 1). Pearson correlation was used as distance measure and average linkage as a linkage method. Since the combination of both ion modes results in redundancy by compounds giving rise to several features, each mode was processed separately. Consistent clusters between the ESI(−) and ESI(+) mode were coloured.

Unspecific signals were more pronounced (87% vs. 75%) in ESI(+) vs. ESI(−). This had led to us to focus on ESI(−) in subsequent analyses.

### Sequence analysis

Genetic distances were estimated from the variant tables available from the 19 genomes project^22^. Loci were reduced to CDS as annotated by the R packages Bsgenome.Athaliana.TAIR.TAIR9^42^ and Genomic Ranges version 1.14.4^43^. For each variant locus, 19 × 19 comparisons were conducted. In order to construct a distance matrix, mismatches were penalized by increasing the distance by 1. The sum of matrices over all 6,400,466 loci was used as a distance matrix (Supplementary Table S2) for hierarchical clustering via the hclust package with average linkage.

Predicted amino acid sequences were processed with BioPerl (Bio::Tools::Run::Alignment::Clustalw, Bio::SeqIO, Bio::Seq, and Bio::AlignIO) and aligned with the Clustalw algorithm with ktuple = 2 and a BLOSUM scoring matrix. Multiple sequence alignments were evaluated for premature ending with the R packages Biostrings version 2.30.1 and plyr version 1.8.1.

### Combination of metabolic and genetic patterns

A metabolic feature was defined as absent when below the limit of detection in all replicates of an accession. Applying this stringent definition, the peak list created from aligning all spectra from ESI(−) was screened for metabolic features with absence, thus reducing the number of features by 25% for exudates ESI(−). The distribution of absence across the 19 accessions is referred to as a pattern. The length of a pattern is the number of accessions that lack the same feature, i.e. a feature absent in Can-0 und Zu-0 is a pattern of length two. Out of the 455 metabolic features in the exudate data set (ESI(−)), 384 were missing in at least one accession. 46 were missing in exactly one accession (length = 1), 52 were absent in two accessions (length = 2) (see Supplementary Table S4). The R package CAMERA version 1.23.2^44^ was used for annotation of adduct species and isotope information. In order to find an association between metabolic patterns of absence and its genetic background, features with a pattern of absence, a monoisotopic annotation by CAMERA and a minimal median intensity of 10,000 were evaluated. 31 features that passed the intensity threshold were matched with stop codon patterns resulting in 9/7/1 features of absence with length 1/2/3.

These matching features or their corresponding quasi-molecular ion were subjected to fragmentation by MS/MS with 10, 20 and 30 eV. Stop codon patterns were derived from multiple sequence alignments of AraCyc enzyme genes^45^ (ftp.plantcyc.org/Pathways/BLAST_sets/aracyc_enzymes.fasta, Dec 2015) as annotated by TAIR10_functional annotations from TAIR.org^46^.

## Additional Information

**How to cite this article**: Mönchgesang, S. *et al*. Natural variation of root exudates in *Arabidopsis thaliana*-linking metabolomic and genomic data. *Sci. Rep.* **6**, 29033; doi: 10.1038/srep29033 (2016).

## Supplementary Material

Supplementary Information
