# Supplementary material for: Natural variation of root exudates in Arabidopsis thaliana-linking metabolomic and genomic data
Source: Sci Rep. 2016 Jul 1;6:29033. doi: 10.1038/srep29033 (PMC4929559; doi:10.1038/srep29033)
Supplement: Supplementary Information [file srep29033-s1.pdf]

Natural variation of root exudates in *Arabidopsis thaliana* - linking metabolomic and genomic data

Susann Mönchgesang[+], Nadine Strehmel[+], Stephan Schmidt[+], Lore Westphal, Franziska Taruttis, Erik Müller, Siska Herklotz, Steffen Neumann and Dierk Scheel*

[+] These authors contributed equally to the manuscript

Supplementary Information

Supplementary Figures 2
Supplementary Tables 4
Supplementary Methods 18

Supplementary Figures

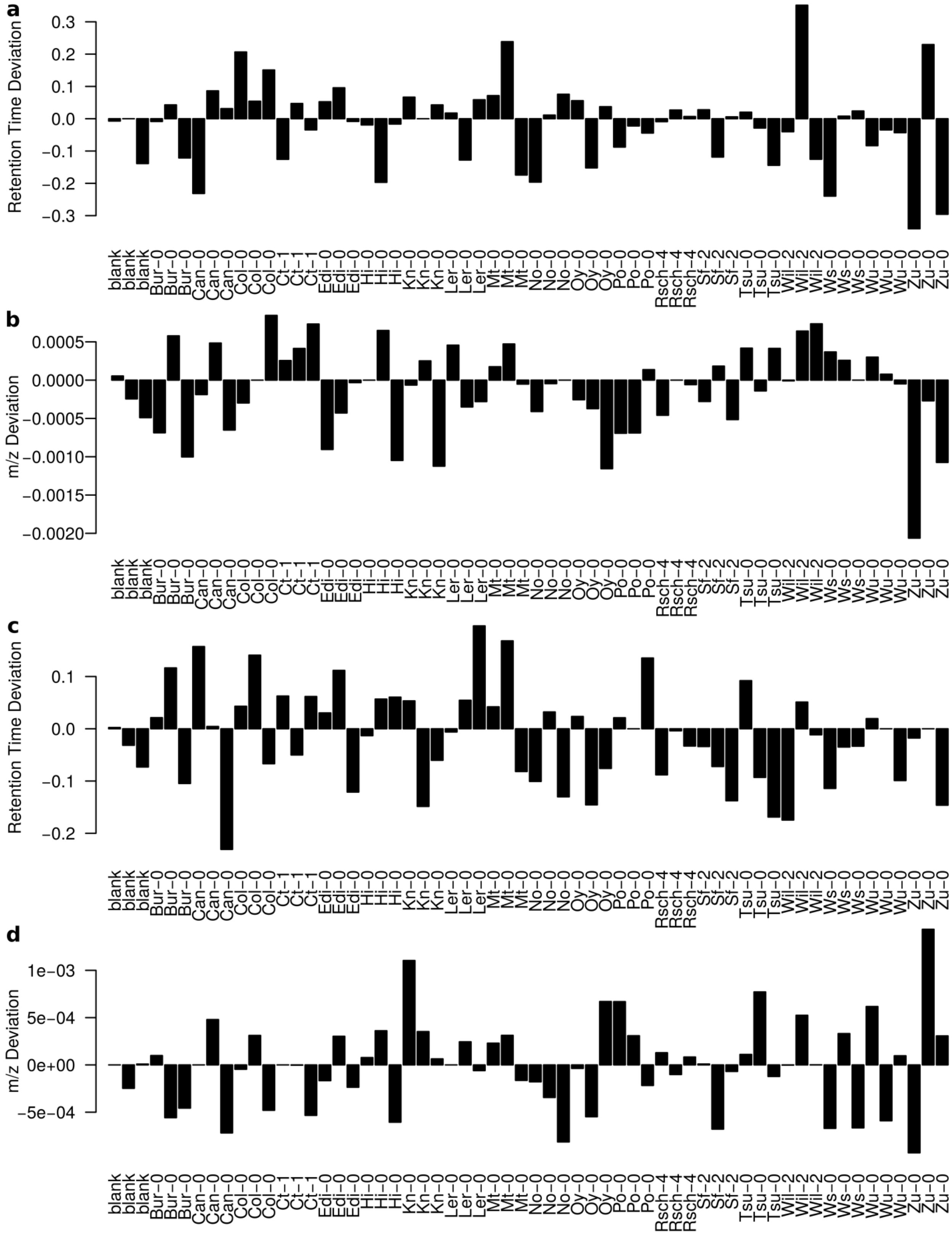

Fig. S1: Quality control of LC/ ESI(-) and ESI(+) MS data sets. Deviation of each sample in chromatography and mass analyzer, (a) *m/z* deviation ESI(-), (b) RT deviation ESI(-), (c) *m/z* deviation ESI(+), (d) RT deviation ESI(+), outlying samples of Oy-0, Ws-0, Hi-0 and Rsch-4 (ESI(+)) did not show greater mass and retention time deviations than others.

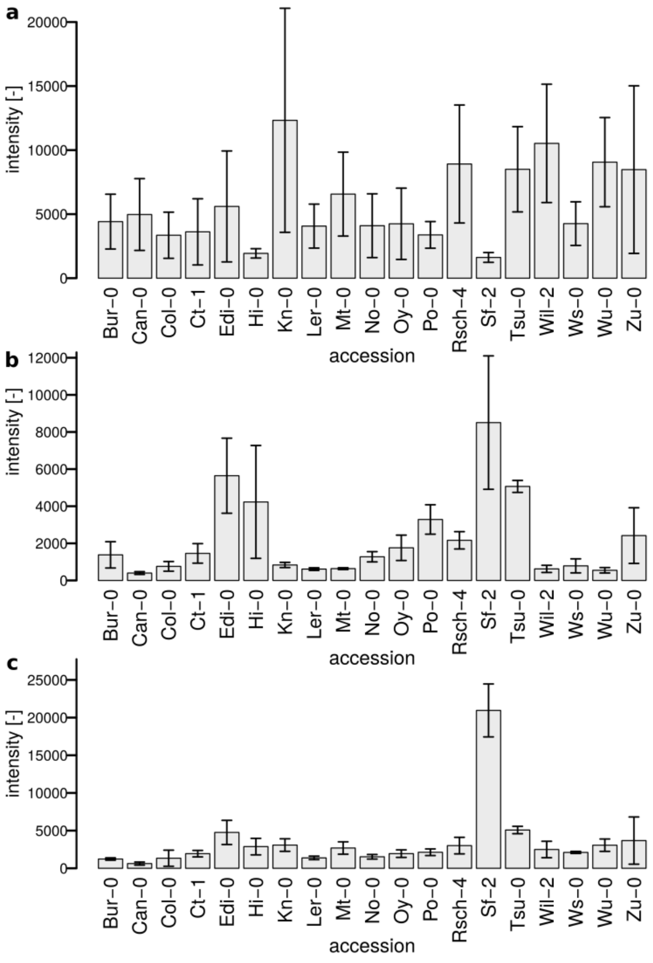

Fig. S2: Intensity distribution of salicylic acid and DHBA hexosides in exudates. a) Salicylic acid, b) 2,5-DHBA hexose, c) 2,3-DHBA hexose, mean ± s.e.m. (n=3).

Supplementary Tables

Table S1: List of *Arabidopsis thaliana* accessions used for the analysis.

Native Name	Short Name	Region
Burren	Bur-0	Ireland
Canary Islands	Can-0	Spain
Catania	Ct-1	Italy
Columbia	Col-0	USA
Edinburgh	Edi-0	UK
Hilversum	Hi-0	Netherlands
Kaunas	Kn-0	Lithuania
Landsberg	L*er*-0	Poland
Martuba	Mt-0	Libya
Nossen	No-0	Germany
Oystese	Oy-0	Norway
Poppelsdorf	Po-0	Germany
Rschew	Rsch-4	Russia
San Feliu	Sf-2	Spain
Tsushima	Tsu-0	Japan
Wilna	Wil-2	Lithuania
Wassilewskija	Ws-0	Russia
Würzburg	Wu-0	Germany
Zürich	Zu-0	Switzerland

Table S2: Distance matrix of genetic differences in coding sequences for hierarchical clustering.

	Bur-0	Can-0	Col-0	Ct-1	Edi-0	Hi-0	Kn-0	Ler-0	Mt-0	No-0	Oy-0	Po-0	Rsch-4	Sf-2	Tsu-0	Wil-2	Ws-0	Wu-0	Zu-0
Bur-0	0	1030565	792507	887818	857745	903915	917335	887489	860940	885751	848296	919135	837762	915748	893187	921528	909193	852591	880827
Can-0	1030565	0	928968	1025039	1019780	1045286	1034321	1029851	1012513	1018098	1001362	1087782	996602	1045749	1015499	1044000	1039973	1011210	1003518
Col-0	792507	928968	0	730260	726645	702147	749298	753504	687807	717405	710378	756093	677352	811442	717691	769821	757255	694172	736591
Ct-1	887818	1025039	730260	0	850940	835285	821301	817589	800136	755504	826759	902903	802428	894552	813927	817694	761791	811764	828838
Edi-0	857745	1019780	726645	850940	0	865699	875206	852735	834757	855075	816516	908182	819374	886218	831016	881231	864960	825039	830550
Hi-0	903915	1045286	702147	835285	865699	0	820651	858658	810845	825608	855132	887875	809976	909813	826070	879506	856821	818618	844396
Kn-0	917335	1034321	749298	821301	875206	820651	0	780892	803193	784265	842836	926208	825612	936144	848379	757938	799659	847400	855314
Ler-0	887489	1029851	753504	817589	852735	858658	780892	0	789252	800336	820920	897880	803782	913381	819941	781239	817647	824538	849352
Mt-0	860940	1012513	687807	800136	834757	810845	803193	789252	0	797971	803262	875480	781386	905338	806286	800381	803746	801273	834739
No-0	885751	1018098	717405	755504	855075	825608	784265	800336	797971	0	806139	875707	794913	901517	794377	806180	789514	806166	829051
Oy-0	848296	1001362	710378	826759	816516	855132	842836	820920	803262	806139	0	548848	787187	891637	809578	849587	824645	768848	831278
Po-0	919135	1087782	756093	902903	908182	887875	926208	897880	875480	875707	548848	0	838695	957886	868491	943971	912028	834712	893581
Rsch-4	837762	996602	677352	802428	819374	809976	825612	803782	781386	794913	787187	838695	0	877323	765640	837435	819634	764630	805938
Sf-2	915748	1045749	811442	894552	886218	909813	936144	913381	905338	901517	891637	957886	877323	0	886189	947490	915272	877277	846368
Tsu-0	893187	1015499	717691	813927	831016	826070	848379	819941	806286	794377	809578	868491	765640	886189	0	847746	832191	805769	816208
Wil-2	921528	1044000	769821	817694	881231	879506	757938	781239	800381	806180	849587	943971	837435	947490	847746	0	820101	869664	880695
Ws-0	909193	1039973	757255	761791	864960	856821	799659	817647	803746	789514	824645	912028	819634	915272	832191	820101	0	831098	861900
Wu-0	852591	1011210	694172	811764	825039	818618	847400	824538	801273	806166	768848	834712	764630	877277	805769	869664	831098	0	812987
Zu-0	880827	1003518	736591	828838	830550	844396	855314	849352	834739	829051	831278	893581	805938	846368	816208	880695	861900	812987	0

Table S3: Compilation of quantifier ions used for the targeted analysis of root exudates. MS identification levels and abbreviations can be found in the original publication by Strehmel *et al.*[1], metabolites indistinguishable from their structural isomers eluting with double peaks are indicated by retention times (RT) separated as RT1/RT2.

ID	NAME	mode	m/z	RT [min]
1	Adenosine	Pos	268.10	0.7
2	Guanosine	Neg	282.08	1.1
3	Uridine	Neg	243.06	1.0
4	2´-Deoxyadenosine	Pos	252.11	0.8
5	Thymidine	Neg	241.08	1.6
6	2´-*O*-Methyladenosine	Pos	282.12	1.1
7	Phe	Pos	120.08	1.3
8	Tyr	Pos	182.08	0.7
9	Trp	Pos	188.07	2.4
10	3-Carboxy-1,2,3,4-tetrahydro-beta-carboline	Pos	144.08	3.1
11	Pentahomo-Met	Pos	174.13	4.6
12	Hexahomo-Met	Pos	188.15	5.4
13	Pentahomo-Met S-Oxide	Pos	190.12	1.4
14	Hexahomo-Met S-Oxide	Pos	204.14	2.7
15	H-Phe-Gly-OH	Pos	223.11	1.7
16	H-Gly-Phe-OH	Pos	223.11	2.3
17	H-Ile-Val-OH	Pos	231.17	1.9
18	H-Val-Ile-OH	Pos	231.17	2.3
19	H-Leu-Val-OH	Pos	231.17	2.3
20	H-Val-Leu-OH	Pos	231.17	2.7
21	H-Leu-Pro-OH	Pos	229.15	2.6
22	H-Leu-Tyr-OH	Pos	295.17	2.8
23	H-Tyr-Ile-OH	Pos	295.17	2.9
24	H-Tyr-Leu-OH	Pos	295.17	3.2
25	H-Phe-Val-OH	Pos	265.15	3.0
26	H-Val-Phe-OH	Pos	265.15	3.3
27	H-Ile-Ile-OH	Pos	245.18	3.1
28	H-Leu-Ile-OH	Pos	245.18	3.3

29	H-Ile-Leu-OH	Pos	245.18	3.4
30	H-Leu-Leu-OH	Pos	245.18	3.6
31	H-Phe-Ile-OH	Pos	279.17	3.8
32	H-Ile-Phe-OH	Pos	279.17	3.9
33	H-Phe-Leu-OH	Pos	279.17	4.1
34	H-Leu-Phe –OH	Pos	279.17	4.2
35	7-MeSO-Heptyl-CN	Pos	210.09	4.4
36	8-MeSO-Octyl-CN	Pos	224.11	5.4
37	8-MeSO-Octyl-NCS	Pos	256.08	8.1
38	8-MeSO-Octyl-CO_2H	Pos	203.11	4.9
39	8-MeSO-Octyl-$CONH_2$	Pos	242.12	3.9
40	7-MeSO-Heptyl-NH_2	Pos	178.12	1.0
41	8-MeSO-Octyl-NH_2	Pos	192.14	2.2
42	8-MeS-Octyl-NH_2	Pos	176.15	5.2
43	Indol-3-ylmethylamine (I3CH_2NH_2)	Pos	130.07	1.8
44	4-MeO-$I_3CH_2NH_2$	Pos	160.08	3.2
45	1-MeO-I3CH_2NH_2	Pos	160.08	3.7
46	Indole-3-carbaldehyde (I3CHO)	Neg	144.04	4.8
47	4-HO-I3CHO	Neg	160.04	5.1
48	Indole-3-carboxylic acid (I3CO_2H)	Pos	118.06	4.7
49	6-(Malonyl-GlcO)-I3CO_2H	Neg	380.10	3.3
50	unknown indole derivative	Pos	174.05	6.5
51	2,3-DHBA 3-O-Xyl	Neg	285.06	3.0
52	2,5-DHBA Pent	Neg	285.06	2.7
53	9,10-Dihydrohydroxy-JA Sulfate	Neg	307.08	3.7
54	Coniferin	Pos	365.12	3.0
55	Syringin	Pos	395.13	3.2
56	G(8-O-4)G	Neg	327.12	4/4.7
57	G(8-O-4)S Glycerol	Neg	439.16	3.2
58	G(8-5)G	Neg	339.12	5.7
59	S(8-5)G	Neg	357.13	5.6
60	S(8-8)S	Neg	417.15	6.3
61	Lariciresinol	Neg	329.14	5.6
62	Lariciresinol Hex	Neg	521.20	4.5
63	G(8-O-4)S(8-5)G	Neg	583.22	6.1/6.3
64	G(8-O-4)S(8-8)S	Neg	613.23	6.7/6.9
65	G(8-O-4)G(8-8)S/G(8-O-4)S(8-8)G	Neg	583.22	6.8/7
66	Esculetin	Neg	177.02	3.4
67	Scopoletin	Pos	193.05	4.5
68	Esculin	Neg	339.07	2.9
69	Scopolin	Neg	191.03	3.3

70	Scopoletin Hex-Pent	Neg	485.13	3.3
71	Scopoletin Benzoyl-Hex-Pent	Neg	589.16	5.5
72	G(8-O-4)Scopoletin	Neg	191.03	4.6
73	G(8-O-4)Esculetin	Neg	355.08	6.3
74	S(8-O-4)Esculetin	Neg	385.09	6.1
75	Scopoletin dehydrodimer	Neg	381.06	5.3
76	Unknown oligolignol	Neg	857.25	5.0
77	G(8-O-4)FA Sulfate	Neg	469.08	4/4.3
78	G(8-5)FA	Neg	353.10	5.9
79	G(8-5)FA Sulfate	Neg	451.07	5.4
80	FA dimer	Neg	387.11	4/4.2
81	Didehydro-di(coumaroyl)spermidine Sulfate	Neg	514.17	3.7
82	Kaempferol 3-O-Rha(1-2)Glc 7-O-Rha	Neg	739.21	3.8
83	9,12,13-Trihydroxy-10(E),15(Z)-octadecadienoic acid	Neg	327.22	7.0
84	9,12,13-Trihydroxyoctadec-10-enoic acid	Neg	329.23	7.4
85	Fatty acid derivative	Neg	309.20	10.6
86	Azelaoyl lyso PC	Neg	426.19	3.9/4.1
87	$C_4H_{10}O$ Hex	Pos	259.11	2.8
88	$C_4H_{10}O$ Malonyl-Hex	Pos	345.11	3.9
89	$C_4H_{10}O$ Hex-DeoxyHex	Neg	381.18	3.4
90	$C_4H_{10}O$ Malonyl-Hex-DeoxyHex	Neg	423.19	4.2
91	$C_7H_9N_5O$ Hex	Neg	340.12	1.2
92	$C_9H_{10}O_3$ Hex	Neg	327.11	3.3
93	$C_{12}H_{16}O_5$ Hex	Neg	401.14	3.4
94	$C_7H_{14}O_4$ Malonyl-Hex	Neg	365.14	3.5
95	$C_{12}H_{16}O_6$ Hex	Pos	441.14	4.5/4.7
96	Unknown	Pos	279.08	5.4/5.6
97	$C_{14}H_{28}O_5$ Malonyl-Hex	Neg	479.25	6.7
98	$C_{18}H_{19}N_5O_7$ Sulfate	Neg	496.08	3.9
99	Unknown secondary amine	Pos	122.09	1.1
100	Unknown	Neg	443.16	3.9
101	Unknown	Neg	471.15	3.1
102	Unknown	Neg	237.04	4.3
103	Unknown	Pos	445.33	9.6
-	Robinin	Neg	739.21	4.3
-	Salicylic acid	Neg	137.02	4.9
-	2,3-DHBA Hexose	Neg	315.07	2.0
-	2,5-DHBA Hexose	Neg	315.07	1.5

Table S4: Absence frequency in exudates ESI(-).

pattern length	occurrence	unique patterns
1	46	13
2	52	32
3	33	30
4	21	20
5	45	43
6	29	27
7	22	21
8	25	24
9	23	23
10	18	18
11	11	11
12	17	17
13	10	10
14	10	9
15	10	9
16	7	6
17	5	2
18	-	
19	-	

Table S5: Mass spectral information of compounds being absent in at least one accession and showing a match with a stop codon of AraCyc enzyme encoding genes.

m/z	RT [min]	adduct	metabolite annotation	collision energy	Fragment ions upon CID m/z (rel. Int. [%], elemental composition)	absent accessions	Gene	gene names
193.05	3.0	[M-H-$C_6H_{10}O_5$]$^-$	Ferulic acid Hex	20 eV	193.05 (100, $C_{10}H_9O_4^-$), 178.02 (53, $C_9H_6O_4^{-}\cdot$), 149.06 (59, $C_9H_9O_2^-$), 134.03 (96, $C_8H_6O_2^{-}\cdot$)	Ct-1	AT1G16780.1	Inorganic H pyrophosphatase family protein
							AT1G22380.1	UDP-glucosyl transferase 85A3
							AT1G53770.2	O-fucosyltransferase family protein
							AT1G66235.1	
							AT1G69720.1	heme oxygenase 3
							AT2G29150.1	NAD(P)-binding Rossmann-fold superfamily protein
							AT3G01010.1	no_Descr
							AT3G26230.1	no_Descr
							AT3G26270.1	no_Descr
							AT3G29630.1	UDP-Glycosyltransferase superfamily protein
							AT3G49630.1	2-oxoglutarate (2OG) and Fe(II)-dependent oxygenase superfamily protein
							AT4G16730.1	terpene synthase 02
							AT4G34930.1	PLC-like phosphodiesterases superfamily protein
							AT5G07440.1	glutamate dehydrogenase 2
							AT5G09910.1	Ras-related small GTP-binding family protein
							AT5G43450.1	2-oxoglutarate (2OG) and Fe(II)-dependent oxygenase superfamily protein
395.20	7.0	[M-2H+Na+CH_2O_2]$^-$	9,12,13-Trihydroxy-10(E),15(Z)-octadecadienoic acid	20 eV	327.22 (16, $C_{18}H_{31}O_5^-$), 291.19 (6, $C_{18}H_{27}O_3^-$), 239.12 (5, $C_{13}H_{19}O_4^-$), 229.14 (57, $C_{12}H_{21}O_4^-$), 221.11 (16,	Ct-1	AT1G16780.1	Inorganic H pyrophosphatase family protein
							AT1G22380.1	UDP-glucosyl transferase 85A3
							AT1G53770.2	O-fucosyltransferase family protein
							AT1G66235.1	
							AT1G69720.1	heme oxygenase 3
							AT2G29150.1	NAD(P)-binding Rossmann-fold superfamily protein
							AT3G01010.1	no_Descr
							AT3G26230.1	no_Descr

					$C_{13}H_{17}O_3^-$), 211.13 (100, $C_{12}H_{19}O_3^-$), 207.10 (3, $C_{12}H_{15}O_3^-$), 193.12 (3, $C_8H_{17}O_5^-$), 183.14 (14, $C_{11}H_{19}O_2^-$), 171.10 (14, $C_9H_{15}O_3^-$), 127.11 (4, $C_8H_{15}O^-$)		AT3G26270.1	no_Descr
							AT3G29630.1	UDP-Glycosyltransferase superfamily protein
							AT3G49630.1	2-oxoglutarate (2OG) and Fe(II)-dependent oxygenase superfamily protein
							AT4G16730.1	terpene synthase 02
							AT4G34930.1	PLC-like phosphodiesterases superfamily protein
							AT5G07440.1	glutamate dehydrogenase 2
							AT5G09910.1	Ras-related small GTP-binding family protein
							AT5G43450.1	2-oxoglutarate (2OG) and Fe(II)-dependent oxygenase superfamily protein
537.21	6.5	[M-2H+Na]-	unknown	10eV	no interpretable MS2 spectrum extractable	Ct-1	AT1G16780.1	Inorganic H pyrophosphatase family protein
							AT1G22380.1	UDP-glucosyl transferase 85A3
							AT1G53770.2	O-fucosyltransferase family protein
							AT1G66235.1	
							AT1G69720.1	heme oxygenase 3
							AT2G29150.1	NAD(P)-binding Rossmann-fold superfamily protein
							AT3G01010.1	no_Descr
							AT3G26230.1	no_Descr
							AT3G26270.1	no_Descr
							AT3G29630.1	UDP-Glycosyltransferase superfamily protein
							AT3G49630.1	2-oxoglutarate (2OG) and Fe(II)-dependent oxygenase superfamily protein
							AT4G16730.1	terpene synthase 02
							AT4G34930.1	PLC-like phosphodiesterases superfamily protein
							AT5G07440.1	glutamate dehydrogenase 2
							AT5G09910.1	Ras-related small GTP-binding family protein
							AT5G43450.1	2-oxoglutarate (2OG) and Fe(II)-dependent oxygenase superfamily protein
301.20	6.3	[M-H]-	unknown	10eV	301.03 (100, $C_{15}H_9O_7^-$), 178.99 (8, $C_8H_3O_5^-$), 151.00 (7, $C_7H_3O_4^-$)	Edi-0	AT1G20132.1	GDSL-like Lipase/Acylhydrolase superfamily protein
							AT1G30710.1	FAD-binding Berberine family protein
							AT1G58260.1	cytochrome p450 79c2
							AT3G42628.1	phosphoenolpyruvate carboxylase-related / PEP carboxylase-related
							AT3G61040.1	cytochrome P450, family 76, subfamily C,

								polypeptide 7
							AT3G62000.2	S-adenosyl-L-methionine-dependent methyltransferases superfamily protein
							AT4G03930.1	Plant invertase/pectin methylesterase inhibitor superfamily
							AT4G20910.1	double-stranded RNA binding protein-related / DsRBD protein-related
							AT4G22350.2	Ubiquitin C-terminal hydrolases superfamily protein
							AT5G07000.1	sulfotransferase 2B
							AT5G20340.1	beta-1,3-glucanase 5
							AT5G22410.1	root hair specific 18
							AT5G24950.1	cytochrome P450, family 71, subfamily A, polypeptide 15
							AT5G28310.1	NAD(P)-binding Rossmann-fold superfamily protein
							AT5G37970.1	S-adenosyl-L-methionine-dependent methyltransferases superfamily protein
							AT5G44440.1	FAD-binding Berberine family protein
							AT5G56680.1	Class II aminoacyl-tRNA and biotin synthetases superfamily protein
439.08	0.5	injection peak	unknown	10eV	no interpretable MS2 spectrum extractable	No-0	AT1G13430.1	sulfotransferase 4C
							AT1G26420.1	FAD-binding Berberine family protein
							AT1G53390.1	P-loop containing nucleoside triphosphate hydrolases superfamily protein
							AT1G64910.1	UDP-Glycosyltransferase superfamily protein
							AT1G64920.1	UDP-Glycosyltransferase superfamily protein
							AT1G72990.1	beta-galactosidase 17
							AT2G46750.1	no_Descr
							AT3G04030.3	no_Descr
							AT3G50300.1	HXXXD-type acyl-transferase family protein
							AT3G56700.1	fatty acid reductase 6
							AT4G09740.1	glycosyl hydrolase 9B14
							AT4G12310.1	cytochrome P450, family 706, subfamily A, polypeptide 5
							AT4G15870.1	terpene synthase 1
							AT4G29580.2	Cytidine/deoxycytidylate deaminase family protein
							AT5G53600.1	Carbohydrate-binding X8 domain superfamily protein

							AT5G67090.1	Subtilisin-like serine endopeptidase family protein
389.12	4.6	[M-H]-	G(8-O-4)FA	10 eV	389.13 (20, $C_{20}H_{21}O_8^-$), 341.10 (100, $C_{19}H_{17}O_6^-$), 282.09 (4, $C_{17}H_{14}O_4^{-\cdot}$), 195.06 (56, $C_{10}H_{11}O_4^-$), 193.05 (99, $C_{10}H_9O_4^-$), 181.05 (2, $C_9H_9O_4^-$), 178.02 (3, $C_9H_6O_4^{-\cdot}$), 165.05 (21, $C_9H_9O_3^-$)	Oy-0	AT1G30740.1	FAD-binding Berberine family protein
							AT1G48910.1	Flavin-containing monooxygenase family protein
							AT2G04440.1	MutT/nudix family protein
							AT2G23510.1	spermidine disinapoyl acyltransferase
							AT2G29320.1	NAD(P)-binding Rossmann-fold superfamily protein
							AT2G31550.1	no_Descr
							AT4G15280.1	UDP-glucosyl transferase 71B5
							AT4G24650.1	isopentenyltransferase 4
							AT4G29460.1	Phospholipase A2 family protein
							AT4G38990.1	glycosyl hydrolase 9B16
							AT5G01780.2	2-oxoglutarate-dependent dioxygenase family protein
							AT5G36220.1	cytochrome p450 81d1
							AT5G37920.1	Family of unknown function (DUF577)
							AT5G45020.1	Glutathione S-transferase family protein
							AT5G45910.1	GDSL-like Lipase/Acylhydrolase superfamily protein
							AT5G49740.1	ferric reduction oxidase 7
380.10	3.2	M-H	6-(Malonyl-GlcO)-I3COOH	20 eV	380.10 (74, $C_{17}H_{18}NO_9^-$), 338.09 (7, $C_{15}H_{16}NO_8^-$), 320.08 (6, $C_{15}H_{14}NO_7^-$), 218.05 (3, $C_{11}H_8NO_4^-$), 176.04 (100, $C_9H_6NO_3^-$), 175.03 (13, $C_9H_5NO_3^{-\cdot}$), 132.04 (11, $C_8H_6NO^-$)	Sf-2	AT1G02730.1	cellulose synthase-like D5
							AT1G32480.1	isocitrate dehydrogenase IV
							AT1G34510.1	Peroxidase superfamily protein
							AT1G51210.1	UDP-Glycosyltransferase superfamily protein
							AT1G60270.1	beta glucosidase 6
							AT1G60530.1	Dynamin related protein 4A
							AT1G60980.1	gibberellin 20-oxidase 4
							AT1G62560.1	flavin-monooxygenase glucosinolate S-oxygenase 3
							AT1G65340.1	cytochrome P450, family 96, subfamily A, polypeptide 3
							AT1G66020.1	Terpenoid cyclases/Protein prenyltransferases superfamily protein
							AT1G77780.1	Glycosyl hydrolase superfamily protein
							AT2G01560.1	Plant protein 1589 of unknown function
							AT2G15470.1	Pectin lyase-like superfamily protein
							AT2G38995.1	no_Descr
							AT2G45970.1	no_Descr

							AT3G14540.1	no_Descr
							AT3G15870.1	no_Descr
							AT3G29635.1	HXXXD-type acyl-transferase family protein
							AT3G44050.1	P-loop containing nucleoside triphosphate hydrolases superfamily protein
							AT4G01770.1	rhamnogalacturonan xylosyltransferase 1
							AT4G10540.1	Subtilase family protein
							AT4G13280.1	terpenoid synthase 12
							AT4G15350.1	cytochrome P450, family 705, subfamily A, polypeptide 2
							AT5G13000.1	glucan synthase-like 12
							AT5G23230.1	nicotinamidase 2
							AT5G47380.1	Protein of unknown function, DUF547
							AT5G49190.1	sucrose synthase 2
							AT5G54500.2	flavodoxin-like quinone reductase 1
739.21	4.3	$[M-H]^-$	Robinin	30 eV	739.21 (11, $C_{33}H_{39}O_{19}^-$), 593.15 (100, $C_{27}H_{29}O_{15}^-$), 430.09 (29.9, $C_{21}H_{18}O_{10}^{-}\cdot$), 284.03 (18.8, $C_{15}H_8O_6^{-}\cdot$)	Wu-0	AT1G04710.1	peroxisomal 3-ketoacyl-CoA thiolase 4
							AT1G09480.1	NAD(P)-binding Rossmann-fold superfamily protein
							AT1G17050.1	solanesyl diphosphate synthase 2
							AT1G44980.1	pectin methylesterase 7
							AT1G45332.1	Translation elongation factor EFG/EF2 protein
							AT1G67000.1	Protein kinase superfamily protein
							AT1G76050.2	Pseudouridine synthase family protein
							AT2G19440.1	O-Glycosyl hydrolases family 17 protein
							AT2G19490.1	recA DNA recombination family protein
							AT2G21860.1	violaxanthin de-epoxidase-related
							AT2G22590.1	UDP-Glycosyltransferase superfamily protein
							AT2G30750.1	no_Descr
							AT3G48110.1	glycine-tRNA ligases
							AT4G35150.1	O-methyltransferase family protein
							AT4G37410.1	cytochrome P450, family 81, subfamily F, polypeptide 4
							AT5G03406.1	Class II aaRS and biotin synthetases superfamily protein
							AT5G24900.1	cytochrome P450, family 714, subfamily A, polypeptide 2
							AT5G38750.1	asparaginyl-tRNA synthetase family

							AT5G41250.1	Exostosin family protein
514.17	3.6	$[M-H]^-$	Didehydro-di(coumaroyl)spermidine Sulfate	10 eV	514.16 (100, $C_{25}H_{28}N_3O_7S^-$), 434.20 (13, $C_{25}H_{28}N_3O_4^-$)	Can-0, Ler-0	AT2G25150.1	HXXXD-type acyl-transferase family protein
							AT2G26620.1	Pectin lyase-like superfamily protein
							AT4G15396.1	cytochrome P450, family 702, subfamily A, polypeptide 6
514.17	4.2	[M-H]-	$C_{25}H_{29}N_3O_7S$	10 eV	514.16 (100, $C_{25}H_{28}N_3O_7S^-$), 434.20 (11, $C_{25}H_{28}N_3O_4^-$)	Can-0, Ler-0	AT2G25150.1	HXXXD-type acyl-transferase family protein
							AT2G26620.1	Pectin lyase-like superfamily protein
							AT4G15396.1	cytochrome P450, family 702, subfamily A, polypeptide 6
582.15	3.6	$[M-2H+Na+CH_2O_2]^-$	Didehydro-di(coumaroyl)spermidine Sulfate	10 eV	514.16 (100, $C_{25}H_{28}N_3O_7S^-$), 434.20 (13, $C_{25}H_{28}N_3O_4^-$)	Can-0, Ler-0	AT2G25150.1	HXXXD-type acyl-transferase family protein
							AT2G26620.1	Pectin lyase-like superfamily protein
							AT4G15396.1	cytochrome P450, family 702, subfamily A, polypeptide 6
473.17	3.4	$[M-H]^-$	$C_{21}H_{30}O_{12}$	20 eV	473.16 (13, $C_{21}H_{29}O_{12}^-$), 235.12 (100, $C_{10}H_{19}O_6^-$), 181.05 (16, $C_9H_9O_4^-$), 137.02 (10, $C_7H_5O_3^-$)	Can-0, No-0	AT1G13430.1	sulfotransferase 4C
							AT1G64920.1	UDP-Glycosyltransferase superfamily protein
433.15	5.5	[M-H]-	$C_{22}H_{26}O_9$	20 eV	433.14 (27, $C_{22}H_{25}O_9^-$), 418.12 (54, $C_{21}H_{22}O_9^{-\cdot}$), 403.1 (34, $C_{20}H_{19}O_9^-$), 373.12 (100, $C_{20}H_{21}O_7^-$), 358.1 (31, $C_{19}H_{18}O_7^{-\cdot}$), 207.07 (9, $C_{11}H_{11}O_4^-$), 193.05 (28, $C_{10}H_9O_4^-$)	Ct-1, Sf-2	AT1G19540.1	NmrA-like negative transcriptional regulator family protein
							AT1G65630.1	DegP protease 3

465.21	7.4	$[M-3H+2Na+2FA]^-$	9,12,13-Trihydroxyoctadec-10-enoic acid	20 eV	329.23 (25, $C_{18}H_{33}O_5^-$), 311.22 (3, $C_{18}H_{31}O_4^-$), 293.21 (2, $C_{18}H_{29}O_3^-$), 229.14 (50, $C_{12}H_{21}O_4^-$), 211.13 (100, $C_{12}H_{19}O_3^-$), 209.12 (4, $C_{12}H_{17}O_3^-$), 193.12 (4, $C_{12}H_{17}O_2^-$), 183.14 (7, $C_{11}H_{19}O_2^-$), 171.1 (19, $C_9H_{15}O_3^-$), 139.11 (7, $C_9H_{15}O^-$), 127.11 (3, $C_8H_{15}O^-$)	Edi-0, Zu-0	AT5G55360.1	MBOAT (membrane bound O-acyl transferase) family protein
473.05	5.4	$[M-H]^-$	$C_{21}H_{30}O_{12}$	20 eV	473.16 (13, $C_{21}H_{29}O_{12}^-$), 235.12 (100, $C_{10}H_{19}O_6^-$), 181.05 (16, $C_9H_9O_4^-$), 137.02 (10, $C_7H_5O_3^-$)	Kn-0, Wil-2	AT5G37400.1	Family of unknown function (DUF577)

537.16	5.8	[M-H]-	$C_{25}H_{30}O_{13}$	20 eV	537.15 (100, $C_{25}H_{29}O_{13}^{-}$), 425.11 (10, $C_{19}H_{21}O_{11}^{-}$), 281.06 (62, $C_{13}H_{13}O_{7}^{-}$), 223.06 (60, $C_{11}H_{11}O_{5}^{-}$), 179.03 (18, $C_{9}H_{7}O_{4}^{-}$), 161.04 (6, $C_{6}H_{9}O_{5}$-), 137.02 (43, $C_{7}H_{5}O_{3}^{-}$), 113.02 (7, $C_{5}H_{5}O_{3}^{-}$)	Ler-0, Ws-0	AT3G42050.1	vacuolar ATP synthase subunit H family protein
285.06	2.6	[M-H]-	2,5-DHBA Pent	10 eV	285.06 (100, $C_{12}H_{13}O_{8}^{-}$), 153.02 (16, $C_{7}H_{5}O_{4}^{-}$), 152.01 (18, $C_{7}H_{4}O_{4}^{-\cdot}$), 108.02 (5, $C_{6}H_{4}O_{2}^{-\cdot}$)	No-0, Oy-0, Sf-2	AT5G04230.2	phenyl alanine ammonia-lyase 3

Supplementary Methods

Plant Cultivation

Seeds were surface-sterilized with chlorine gas for 40 min and left to stratify over night at 4 °C. All equipment was autoclaved prior to usage and all preparation steps were performed under a laminar flow hood.

For hydroponic cultivation, a pre- and main culture set-up was used. The pre-culture system consisted of PCR tubes filled with approx. 170 µL agar solution (8 % (w/v) GELRITE, 1 % (w/v) sucrose) placed in a yellow pipette tip box. After solidification, bottoms were cut and individual seeds were sown on a total of 48 tubes submerged partially in nutrient solution (half-strength Murashige and Skoog (MS) medium supplemented with 1 % (w/v) sucrose and Gamborg's B-5 vitamins (Duchefa), pH 5.8). Boxes were closed with their lids and sealed with leucoplast (Duchefa). Plants were grown at 22 °C under short-day conditions (8 h light, 130 lmol/m^2/s) to prevent early inflorescences until all roots had penetrated the agar plug. After three weeks, the main-culture system was set up. Tubes were transferred into perforated screw caps of 50mL amber bottles (Duran, Wertheim/Germany) filled with approximately 70 mL nutrient solution. Modified MS medium (1.0 mM KH_2PO_4, 1 mM $MgSO_4$, 0.25 mM K_2SO_4, 0.25 mM $CaCl_2$, 2 mM NH_4-NO_3, 0.1 mM Na-Fe-EDTA, 50 µM KCl, 30 µM H_3BO_3, 5 µM $MnSO_4$,1 µM $ZnSO_4$, 1 µM $CuSO_4$, 0.7 µM $NaMoO_4$, pH 5.8) was used as a culturing medium to enable plant growth over a prolonged period without osmotic stress and sufficient nutrient supply. Medium was exchanged weekly and collected after one-week-exudation at week 6 still in the vegetative stage accompanied by a check for microbial contamination. Glass bottles were kept in sealed plastic boxes (Araponics, Liège/Belgium) with 12 plants each. PCR tubes filled with agar were handled in the same way and used as a blank. Each of the three independent experiments was run with four plants per accession in the main culture whose exudates were pooled.

SALK_088702C was analysed in a small-scale system[2] with one week pre-culture and two weeks main cultivation with the pooled exudates of 16-20 plants (one week exudation).

Sample preparation

Due to the dilution effect in the hydroponic system, root exudates need to be enriched and cleared of excessive inorganic salts from the nutrient solution. 280 mL of pooled plant medium was spiked with 4 µg (2,4-dichlorophenoxy)acetic acid and concentrated until dryness using a vacuum rotary evaporator (40 °C, 250 mL round-bottom flask). The residue was reconstituted in 9 mL 5 % (v/v) methanol and sonicated for 10 min at 20 °C. After transfer to 15 mL falcon tubes, the concentrated solution was centrifuged at 6000xg for 10 min and 4 mL were subjected to solid phase extraction with a Bond Elut

C18 column (500 mg, 3 mL, Agilent) to enrich for the semipolar fraction of root exudates. The cartridge was conditioned with 1 mL methanol, equilibrated with 1 mL 2 % (v/v) formic acid, and upon sample loading washed with 1 mL water. 1 mL 2% (v/v) formic acid in methanol was used to elute retained compounds. 500 µL of the eluate were evaporated to dryness at 40 °C using a vacuum centrifuge. The remaining residue was reconstituted in 60 µL 30% (v/v) methanol and sonicated for 5 min at 20 °C. After centrifugation (16,000xg, 15 min) the supernatant was transferred into a glass vial and subjected to UPLC/ESI-QTOFMS analysis

Non-targeted metabolite profiling analysis

Samples (2.6 µL) were injected via full loop onto an Acquity UPLC system (Waters, Eschborn/Germany) which had been mounted with a HSS T3 column (100 x 1.0 mm, particle size 1.8 µm, Waters), and separated using a binary gradient (flow rate: 150 µL/min); namely 0–1 min, isocratic 95 % A (water/ 0.1 % formic acid), 5 % B (acetonitrile / 0.1 % formic acid); 1–16 min, linear from 5 to 95 % B; 16–18 min, isocratic 95 % B; 18–18.01, linear from 95 to 5 % B; 18.01–20 min, isocratic 5 % B.

Eluting compounds were detected in positive and negative ionization mode from m/z 80 – 1000 using a MicroTOF–Q I hybrid quadrupole time-of-flight mass spectrometer equipped with an Apollo II electrospray ion source (Bruker Daltonics, Billerica/MA, USA). Instrument parameters were defined as follows for the positive mode: nebulizer gas - nitrogen, 1.6 bar; dry gas - nitrogen, 6 L/min, 190 °C; capillary voltage- -5000 V; end plate offset - -500 V; funnel 1 RF – 200 Vpp; funnel 2 RF – 200 Vpp; in-source CID energy – 0 V; hexapole RF – 100 Vpp; quadrupole ion energy- 5 eV, low mass 55; collision gas - argon; collision energy – 10 eV; collision RF – 150 Vpp; transfer time – 70 µs; pre pulse storage – 5 µs; spectra rate – 3 Hz. For the negative ionization mode all parameters were maintained except for the capillary voltage (4000 V).

Data availability

The full data set was made available at the MetaboLights repository under the accession number MTBLS160 (http://www.ebi.ac.uk/metabolights/MTBLS160, private pre-publication-reviewer link http://www.ebi.ac.uk/metabolights/reviewerLgTnoHUrFb). The metadata is available in the ISA (Investigation, Study, Assay) format. The first file, i_Investigation.txt describes the general aims, experimental protocols, instrumentation, authors and relevant publications. The second file, s_Study.txt holds the sample table, and in particular the association between individual hydroponic flasks and pooled exudate samples. Finally, several a_Assay.txt files describe the acquired mass spectrometry data. The resulting metabolite annotation and identification, including the quantification, is in the m_metabolite.tsv files. We also included the Rdata files for the

xcmsSets after the data analysis steps. The data are available under the EBI public license.

DNA and RNA analysis

Nucleic acids were extracted using the RNeasy plant Mini Kit (Qiagen) from frozen roots according to the manufacturer's instructions. DNA was extracted with a quick prep protocol using an ethanol precipitation. Upon DNase digest, RNA was eluted and transcribed into cDNA using Revert-Aid H Minus First-Strand Synthesis Kit (Fermentas) from 5 µg of total RNA.

The homozygous T-DNA insertion was confirmed by a double reaction PCR with gene-specific primer pairs (N676345_LP TTACGTTTTCAAAGCCAATGG, N676345_RP AAACATGTCGGATTCCAACAG for SALK_098927C; SALK_088702_LP TTAACCGACCATTGGATCAAG, SALK_088702_RP GTTTTGGCTTCCTCCAATCTC) and the T-DNA insertion specific primer *LBb1.3*. ATTTTGCCGATTTCGGAAC. The following thermal cycle protocol was used: 5 min initial denaturation, 45 cycles of 30 s denaturation (95°C), 30 s annealing (52 °C) and 75 s elongation (72 °C) followed by 10 min final elongation (72 °C).

To determine transcript levels of SCT and UGT91A1, quantitative real-time PCR was performed with SYBR green (Thermo) in 10 µL reaction mixtures using a MX3005P cycler (Agilent) and the following primers: At2g25150_fw CTTGAAAAGAAACCAGTTGAGC, At2g25150_rev TTGCCCGAAAGAGGGTAGTAA[3] for SCT and At2g22590_fw GACGAGACAGAAGGTTTCTTTAC, At2g22590_rev CCACATAACGATCTTGTCTATCC[4], ugt91a1_fw2 GACGTCCCTTTCGAACTCAT ugt91a1_rev2 GACGAGAGATTGGAGGAAGC (binding after and before the insertion site) for UGT91A1. Amplification was performed by 10 min initial denaturation (72 °C), 40 cycles of 30 s denaturation (95 °C), 30 s annealing (60 °C) and 30 s elongation (72 °C), final elongation of 10 min (72 °C) and a subsequent dissociation curve measurement (1 min denaturation (95 °C), 30 s annealing (60 °C), 30 s denaturation (95 °C)). PP2A was used as a reference transcript (PP2A_fw CTTGGGAAATTGTTGGAGCC, PP2A_rev CAACCATATAACGCACACGC). Primer efficiency (E) was estimated as exp(-1/m) from the slope m of cycles-to-threshold (ct) values for a dilution series. Relative transcript levels were determined as the ratio of E^{ct} between the reference and gene of interest. Each measurement was performed in duplicate for each sample.

References

1 Strehmel, N., Böttcher, C., Schmidt, S. & Scheel, D. Profiling of secondary metabolites in root exudates of Arabidopsis thaliana. *Phytochemistry*. **108C,** 35-46 (2014).

2 Ziegler, J. *et al.* Non-targeted profiling of semi-polar metabolites in Arabidopsis root exudates uncovers a role for coumarin secretion and lignification during the local response to phosphate limitation. *J Exp Bot*. **67,** 1421-1432 (2016).

3 Luo, J. *et al.* A novel polyamine acyltransferase responsible for the accumulation of spermidine conjugates in Arabidopsis seed. *Plant Cell*. **21,** 318-333 (2009).

4 Stracke, R. *et al.* Differential regulation of closely related R2R3-MYB transcription factors controls flavonol accumulation in different parts of the Arabidopsis thaliana seedling. *Plant J*. **50,** 660-677 (2007).
